# Effects of Gamma-Tocotrienol on Intestinal Injury in a GI-Specific Acute Radiation Syndrome Model in Nonhuman Primate

**DOI:** 10.3390/ijms23094643

**Published:** 2022-04-22

**Authors:** Sarita Garg, Tarun K. Garg, Stephen Y. Wise, Oluseyi O. Fatanmi, Isabelle R. Miousse, Alena V. Savenka, Alexei G. Basnakian, Vijay K. Singh, Martin Hauer-Jensen

**Affiliations:** 1 Division of Radiation Health, Department of Pharmaceutical Sciences, University of Arkansas for Medical Sciences, Little Rock, AR 72205, USA; gargsarita@uams.edu; 2 Department of Biochemistry and Molecular Biology, University of Arkansas for Medical Sciences, Little Rock, AR 72205, USA; iracinemiousse@uams.edu; 3 UAMS Myeloma Center, University of Arkansas for Medical Sciences, Little Rock, AR 72205, USA; gargtarunk@uams.edu; 4 Division of Radioprotectants, Department of Pharmacology and Molecular Therapeutics, F. Edward Hébert School of Medicine, Uniformed Services University of the Health Sciences, Bethesda, MD 20814, USA; stephen.wise.ctr@usuhs.edu (S.Y.W.); oluseyi.fatanmi@usuhs.edu (O.O.F.); vijay.singh@usuhs.edu (V.K.S.); 5 Armed Forces Radiobiology Research Institute, Uniformed Services University of the Health Sciences, Bethesda, MD 20814, USA; 6 Department of Pharmacology and Toxicology, University of Arkansas for Medical Sciences, Little Rock, AR 72205, USA; savenkaalenav@uams.edu (A.V.S.); basnakianalexeig@uams.edu (A.G.B.); 7 John L. McClellan Memorial VA Hospital, Central Arkansas Veterans Healthcare System, Little Rock, AR 72205, USA

**Keywords:** gamma-tocotrienol, intestine, radiation, nonhuman primates, radiation countermeasure

## Abstract

The gastrointestinal (GI) system is highly susceptible to irradiation. Currently, there is no Food and Drug Administration (FDA)-approved medical countermeasures for GI radiation injury. The vitamin E analog gamma-tocotrienol (GT3) is a promising radioprotector in mice and nonhuman primates (NHP). We evaluated GT3-mediated GI recovery in total-body irradiated (TBI) NHPs. Sixteen rhesus macaques were divided into two groups; eight received vehicle and eight GT3 24 h prior to 12 Gy TBI. Proximal jejunum was assessed for structural injuries and crypt survival on day 4 and 7. Apoptotic cell death and crypt cell proliferation were assessed with TUNEL and Ki-67 immunostaining. Irradiation induced significant shortening of the villi and reduced mucosal surface area. GT3 induced an increase in crypt depth at day 7, suggesting that more stem cells survived and proliferated after irradiation. GT3 did not influence crypt survival after irradiation. GT3 treatment caused a significant decline in TUNEL-positive cells at both day 4 (*p* < 0.03) and 7 (*p* < 0.0003). Importantly, GT3 induced a significant increase in Ki-67-positive cells at day 7 (*p* < 0.05). These data suggest that GT3 has radioprotective function in intestinal epithelial and crypt cells. GT3 should be further explored as a prophylactic medical countermeasure for radiation-induced GI injury.

## 1. Introduction

The increasing risk of exposure to ionizing radiation (IR) due to nuclear or radiological incident is a serious concern to civilians as well as the military encountering radiological hazards [1]. Total-body or significant partial-body exposures to IR can result in acute radiation syndrome (ARS) with doses greater than 1 Gy, delivered at relatively high dose rate. One likely outcome of such exposure is death/injury to the rapidly renewing cells such as the bone marrow and gastrointestinal (GI) system [2,3]. Studies indicate that GI damage is a major factor responsible for irradiation-induced lethality in humans receiving high doses of IR [4]. The GI tract is particularly susceptible to radiation exposure due to its continually proliferating cell populations [5,6,7]. Typically, a high dose of radiation induces loss of intestinal stem cells, and thereby, impairs epithelial regeneration [8]. The classical characteristics of GI injury include loss of clonogenic crypt epithelial stem cells, which leads to enterocyte depletion, villus blunting, and damage to the mucosal layer of the small intestine. Diarrhea and mucosal barrier breakdown accompanied by weight loss, dehydration, reduced nutrient absorption, and intestinal bleeding/infection, if not controlled, may lead to sepsis [9]. In severe cases, it may result in death (hallmark of the GI syndrome) [10]. To date, there are no Food and Drug Administration (FDA)-approved medical countermeasures to treat GI-ARS, as all four FDA-approved products for ARS address only hematopoietic (H) injuries [4,11,12,13]. 

Components of vitamin E (tocols; tocopherols, and tocotrienols) are well known for their antioxidative, neuroprotective, anti-inflammatory, and radio-prophylactic properties [14,15,16]. Among the tocols tested to date, gamma-tocotrienol (GT3) has received significant attention during the last 10 years and appears to be one of the most encouraging prophylactic radiation countermeasures for hematopoietic (H-ARS) under investigation [12]. GT3 is of particular interest because, in addition to being an antioxidant, it also inhibits 3-hydoxy-3-methylglutaryl-coenzyme A (HMG-CoA) reductase, the rate limiting enzyme in cholesterol biosynthesis [17,18], and accumulates to a greater extent in endothelial cells [19]. We and others showed that GT3 not only protects against gastrointestinal, vascular, and hematopoietic injury [20,21], but also accelerates recovery of intestinal cells and improves mucosal barrier integrity post-irradiation [22]. The radioprotective efficacy of GT3 in mice is mediated largely via granulocyte colony-stimulating factor (G-CSF), which helps mobilize progenitor cells to protect against hematopoietic injury [23,24,25]. In addition, GT3 modulates the expression of pro-apoptotic and anti-apoptotic genes to promote intestinal stem cell recovery and thus confers protection against radiation-induced intestinal injury [26]. GT3 has been shown to be a radioprotector in the murine model of ARS [24]. Importantly, GT3 has demonstrated its effectiveness as a radioprotector, in promoting hematopoietic recovery in the nonhuman primate (NHP) model [27]. Furthermore, GT3 has been shown to restore IR-induced micro-RNA changes in NHPs [28], and also restore IR-induced proteomic changes back to baseline in the murine model [29].

The present study was undertaken to evaluate GT3 potential to accelerate GI recovery in irradiated NHPs when exposed to a supralethal 12 Gy TBI. We hypothesized that drugs that successfully mitigate radiation damage in the small intestine will influence intestinal stem cell survival and/or proliferation. Results from this study demonstrated that a supralethal total-body radiation dose of 12 Gy induced significant intestinal injury while, GT3 treatment was associated with reduced intestinal apoptosis and enhanced stem cell proliferation. However, GT3 did not influence crypt survival. Taken together, these data suggest that GT3 has a protective role in the intestinal epithelial and crypt cells in the context of irradiation-induced gut damage. GT3 should be further investigated as a potential medical countermeasure for irradiation-induced GI injury.

## 2. Results

### 2.1. Irradiation-Induced Histological and Morphometric Injury in Proximal Jejunum

**Figure 1:**
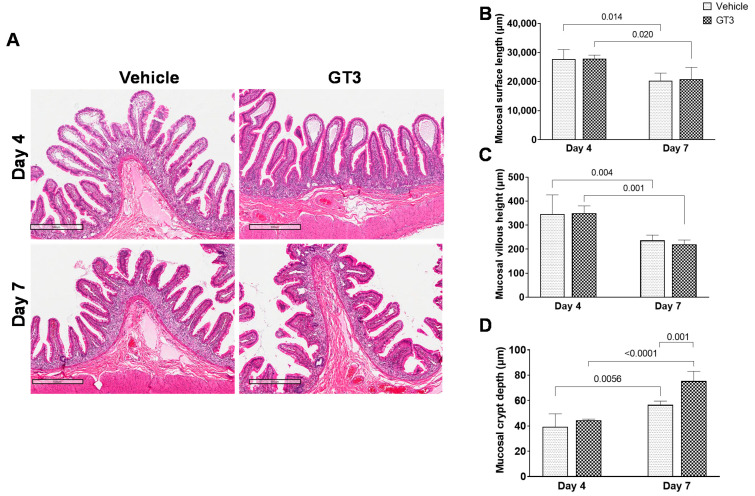
Effects of GT3 on irradiation-induced intestinal damage at day 4 and day 7. (**A**) Representative images showing the cross sections of proximal jejunum treated with vehicle or GT3 at day 4 and 7 post-irradiation. Histogram showing the measurements of intestinal injury such as (**B**) mucosal surface length, (**C**) villous height, and (**D**) crypt depth. The data are presented as average ± SEM; *N* = 3 (day 4) and *N* = 5 (day 7).

The loss of epithelial cells after irradiation shortens/blunts the villi, disrupting the structural integrity of the intestine. Here, we assessed the role of GT3 in modulating irradiation-induced intestinal structural injury in the jejunum sections of GI at day 4 and 7 post-irradiation. As shown in Figure 1A, radiation induced shortening of the villi and decreased mucosal surface length in both vehicle and GT3-treated groups by day 7. Crypt atrophy was observed at both day 4 and day 7.

According to morphometric studies, mucosal surface length was significantly reduced at day 7 in both vehicle (*p* < 0.01) and GT3-treated (*p* < 0.02) groups compared to their respective groups on day 4. However, there was no difference between vehicle- and GT3-treated groups when compared at either day 4 or day 7 (Figure 1B). Likewise, villous height was significantly reduced at day 7 in both vehicle (*p* < 0.004) and GT3-treated groups (*p* < 0.001) when compared to day 4 (Figure 1C), but there was no significant difference noted between the groups on either day 4 or 7. A strong positive correlation has been identified between crypt depth and stem cell survival after irradiation [30]. Interestingly, at day 7, GT3 treatment significantly increased crypt depth when compared to the vehicle group (*p* < 0.001) at day 7 and the GT3-treated group at day 4 (*p* < 0.0001), respectively (Figure 1D). However, at day 4, no differences in crypt depth were noted between the vehicle and GT3-treated groups. However, GT3 showed a significant increase in crypt depth by day 7 (the latest time point examined in this study). 

### 2.2. Effects of Irradiation on Intestinal Crypt Survival

**Figure 2:**
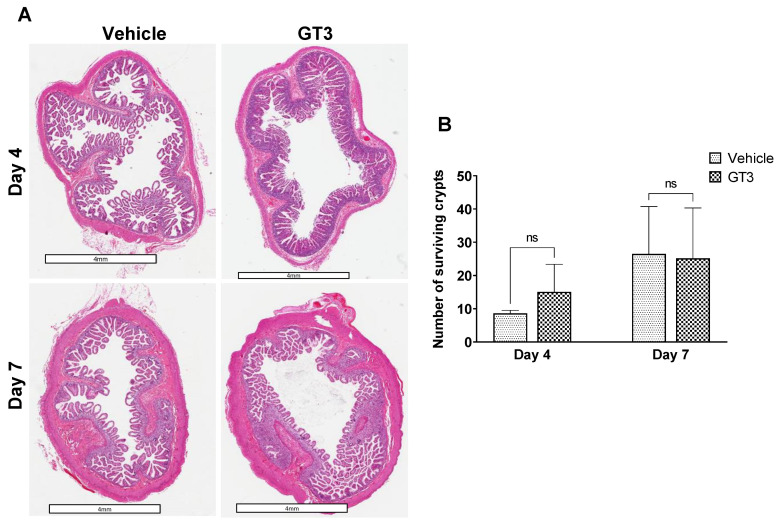
Effects of irradiation on crypt survival. (**A**) Representative images showing the transverse sections of proximal jejunum from NHP treated with vehicle or GT3 at day 4 and 7 post-irradiation. (**B**) Surviving crypts. The data are presented as average ± SEM; *N* = 3 (day 4) and *N* = 5 (day 7). ns = not significant.

Intestinal crypt survival was determined at day 4 and day 7 post-TBI. GT3 treatment did not affect irradiation-induced crypt survival either at day 4 or day 7 post-irradiation (Figure 2A). However, in comparison to vehicle at day 4, GT3-treated group showed an increase in crypt survival, though the level of significance was not achieved (Figure 2B). 

### 2.3. GT3 Inhibited Irradiation-Induced Apoptosis in the Intestinal Jejunum

**Figure 3:**
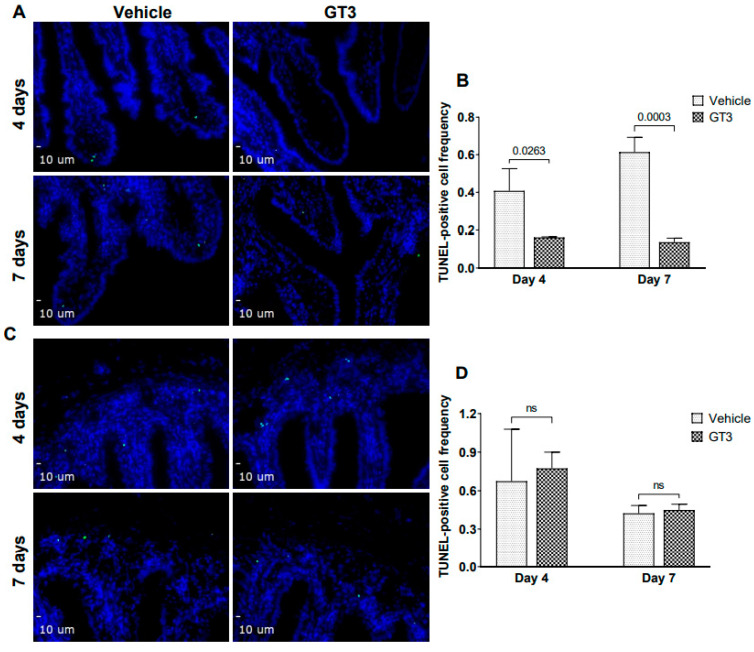
Effects of GT3 on TBI-induced intestinal damage. (**A**) Representative photomicrograph of TUNEL-positive cells (green) in the intestinal villi. (**B**) Frequency of TUNEL-positive cells in villi at day 4 and 7 post-TBI in vehicle and GT3-treated groups. (**C**) Representative photomicrograph of TUNEL-positive cells (green) in the crypt. (**D**) Frequency of TUNEL-positive cells in the crypts at day 4 and 7 post-TBI in vehicle and GT3-treated groups. The data are presented as average ± SEM; *N* = 3 (day 4) and *N* = 5 (day 7). ns = not significant.

Irradiation-induced apoptosis of intestinal crypt epithelial cells and stromal cells in the villus, characterized by DNA fragmentation, is an important contributor to ARS and determines survivability. Apoptotic cell death was assessed by performing TUNEL staining of NHP intestinal tissue sections at days 4 and 7 following irradiation. The intestinal epithelium undergoes rapid cell turnover and is replenished by stem and progenitor cells that reside in the crypt [31]. The transit time varies depending on factors such as region of measurement, diet, etc. Here, we observed a significant decline in the frequency of TUNEL-positive cells in the villi of GT3-treated groups at both day 4 (*p* < 0.03) and day 7 (*p* < 0.0003) post-irradiation (Figure 3A,B). On the contrary, no significant difference was observed in the frequency of TUNEL-positive cells in the crypts of vehicle- or GT3-treated animals at both day 4 and 7 (Figure 3C,D).

### 2.4. GT3 Enhanced Cell Proliferation in the Jejunum Crypts Post-Irradiation

The intestinal epithelium renewal relies on crypt intestinal stem cells and is crucial for re-establishment of the jejunum architecture that becomes compromised post-irradiation [32,33,34]. Ki-67 is a nuclear antigen present in the proliferating cells and is one of the most widely used proliferating cell markers. Here, we determined the effects of GT3 on the crypt cell proliferation at day 4 and 7, by evaluating the expression of Ki-67 in the jejunum exposed to 12 Gy TBI.

**Figure 4:**
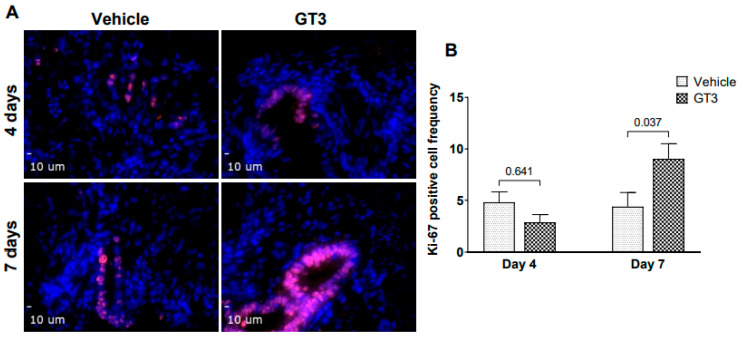
Effects of GT3 on intestinal stem proliferation as assessed by Ki-67. (**A**) Representative photomicrograph of Ki-67-positive cells (red) in the crypts. (**B**) Mean fluorescence intensity of Ki-67 at day 4 and 7 post-TBI in vehicle and GT3-treated groups. The data are presented as average ± SEM; *N* = 3 (day 4) and *N* = 5 (day 7).

**Figure 5:**
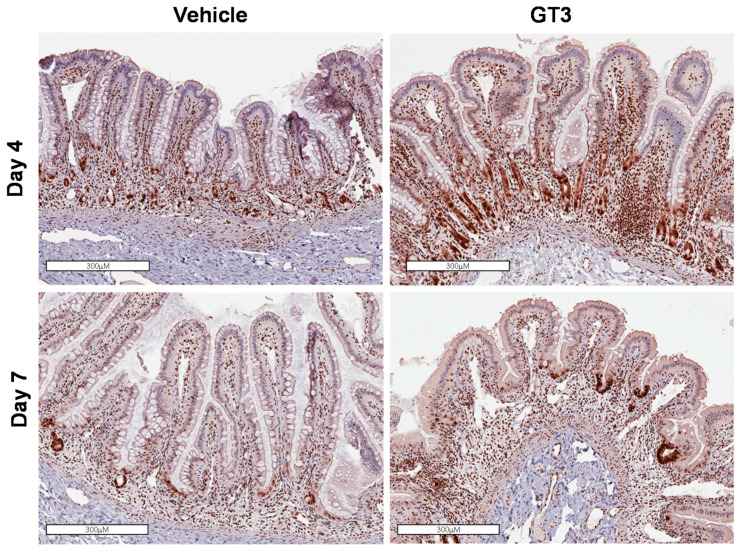
Effects of GT3 on PCNA expression in proximal jejunum post-irradiation. Representative images showing the expression of PCNA in cross sections of proximal jejunum treated with vehicle or GT3 prior to exposure to 12 Gy TBI at day 4 and day 7 post-irradiation.

We observed no difference in the intestinal Ki-67-positive cells at day 4 between vehicle- and GT3-treated groups (Figure 4A). Interestingly, when compared to vehicle, GT3-treated animals showed a significant increase in intestinal Ki-67-positive cells at day 7 post-TBI (*p* < 0.03) (Figure 4B). In addition to Ki-67, we also evaluated the expression of proliferating cell nuclear antigen (PCNA), another critical marker for cell proliferation that plays an essential role in DNA synthesis/replication. Several monoclonal antibodies to PCNA, which show peak expression at the late G1-S phase have been developed to identify the proliferating cells [35,36]. PCNA analysis also revealed an increased expression in proliferating crypts in GT3-treated groups at both day 4 and 7, when compared to their vehicle groups, respectively (Figure 5). 

### 2.5. Effects of GT3 on Irradiation-Induced Alterations in Tight Junction-Related Proteins in the Jejunum

**Figure 6:**
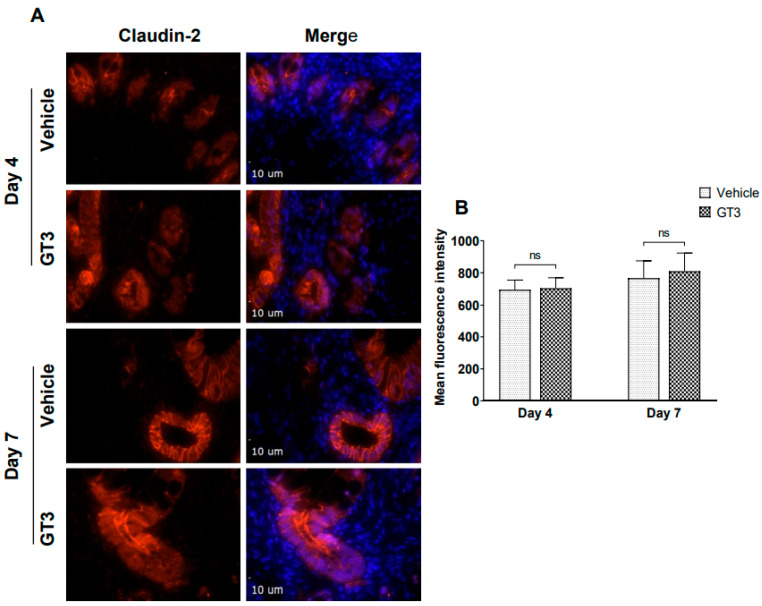
**.** Effects of GT3 on claudin-2 expression in intestine. (**A**) Representative photomicrograph of claudin-2 expression (red) in the cross sections of the intestine at day 4 and day 7 post-irradiation. (**B**) Quantitation of claudin-2 expression levels in intestinal crypts at indicated time points post-12 Gy TBI. The data are presented as average ± SEM; *N* = 3 (day 4) and *N* = 5 (day 7). ns = not significant.

**Figure 7:**
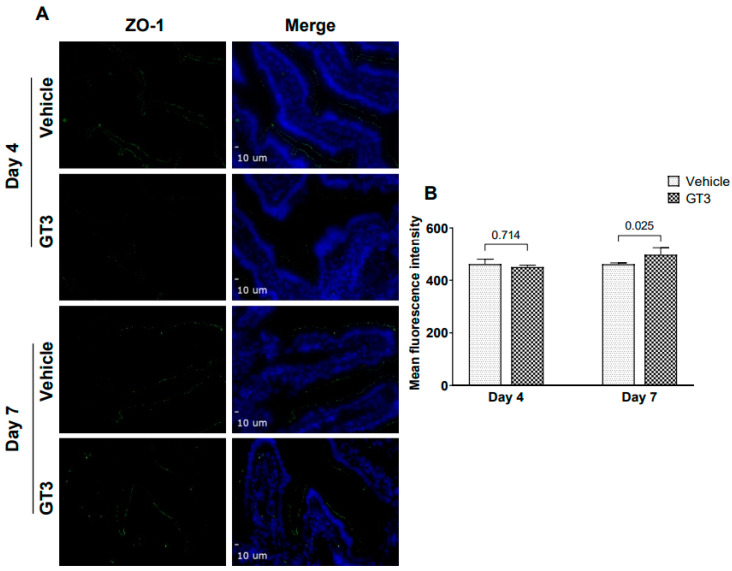
Effects of GT3 on ZO-1 expression in intestine. (**A**) Representative photomicrograph of ZO-1 expression (green) in the cross sections of the intestine at day 4 and day 7 post-irradiation. (**B**) Quantitation of ZO-1 expression levels in intestine at indicated time points post-12 Gy TBI. The data are presented as average ± SEM; *N* = 3 (day 4) and *N* = 5 (day 7).

Studies have shown that radiation injury led to downregulation of the tight junction proteins in the intestinal mucosa [37]. Interestingly, GT3 has been shown to effectively reduce post-TBI increase in gut hyper-permeability in a mouse model [22]. Here we performed immunofluorescence staining by using a panel of TJs-related antibodies to evaluate if GT3 could contribute to the restoration of the mucosal barrier dysfunction induced by radiation. No significant differences were observed in the expression levels of claudin-2, in vehicle- or GT3-treated groups at either day 4 or day 7 (Figure 6). Interestingly, a significant increase was noted in ZO-1 (*p* < 0.02) expression level in the GT3-treated group at day 7 in comparison to vehicle (Figure 7). However, there was no difference noted between the groups at day 4. Furthermore, no significant differences were noted in the expression levels of claudin-7, occludin, beta-catenin, and E-cadherin between vehicle and GT3-treated groups either at day 4 or day 7 (data not shown).

## 3. Discussion

ARS is one of the most challenging aspects of a public health and medical response to a nuclear or radiological incident. The clinical progression of ARS depends on various factors, including the absorbed radiation dose, the intensity of exposure, and its distribution within bodily tissues [38]. Irradiation-induced GI injury constitutes an important element of ARS with life threatening implications and challenges in developing therapeutic interventions [2]. Development of countermeasures targeting amelioration of GI-ARS has been limited by a lack of understanding of the disease sequelae of this syndrome in a large animal model, such as NHP [4]. The NHP has been a useful model for studies of human ARS as NHPs are closest to humans in respect of genetic homology and pathophysiology [39,40,41]. Most importantly, both NHPs and humans exhibit strong similarities in intestinal structure, physiology, immunology, function, and their microbiome. This study evaluated the potential of GT3 in accelerating GI-recovery in NHPs exposed to a dose of 12 Gy total-body radiation.

The concept of operations (CONOPS) developed by the US government agencies demonstrates that it will be difficult to have required treatments or triage devices needed in a time frame earlier than 24 h after radiation exposure. For this reason, the majority of biodosimetry devices and medical countermeasure (MCM) development for civilian use have assumed triage or drug administration at 24 h or later after the incident. Pre-exposure prophylactic agents are of interest to the Department of Defense and are needed for military personnel and first responders. In contrast, post-exposure radiomitigators would be useful for the civilian population who do not know ahead of time about their exposure. GT3 is a promising radiation countermeasure under advanced development which can be used 24 h prior to radiation exposure and is relevant to military and first responders. All four agents approved by US FDA for H-ARS are radiomitigators to be used 24 h or more after radiation exposure

GT3 has been shown to be a promising radioprotector in murine and NHP models [20,27], and was most effective when administered 24 h prior to exposure with a lethal dose of ionizing radiation [12]. Further, a single prophylactic dose of GT3, without any supportive care was equally effective in improving hematopoietic recovery to multiple doses of Neupogen/Leukine and two doses of Neulasta, with full supportive care in the NHP model [27]. In addition, metabolic changes induced by GT3 administration in NHP were associated with a transient increase in the bioavailability of antioxidants, which may provide an overall advantage to combat radiation injury [42]. GT3 is a well-known antioxidant and free radical scavenger [12,43]. In a recent study, we demonstrated restoration of proteomic change in response to cobalt-60 gamma-irradiation using a murine model [29]. GT3 has also been shown to restore irradiation-induced micro-RNA changes in NHP blood 24 h after radiation exposure [28]. Here, we show that radiation exposure to a supralethal dose of 12 Gy total-body induced significant intestinal injury in the form of a reduced mucosal surface area and villus height, while GT3 prophylaxis was associated with increased crypt depth. Notably, GT3 treatment reduced TBI-induced apoptosis in the jejunum and stimulated crypt stem cell proliferation, critical components needed to replenish the damaged epithelium.

Irradiation-induced structural changes in the intestine involve a highly complex pathophysiology, that includes, but not limited to, alterations in redox status, inflammation-related secondary effects, and impaired functionality due to loss of cells [8,44]. Several studies have widely documented irradiation-induced intestinal damage in NHP, rat, and mouse models of partial- and total-body irradiation [5,45,46,47,48,49]. We demonstrated that TBI doses of 6.7 and 7.4 Gy (typically associated with H-ARS) induced significant intestinal injury in a dose and time-dependent manner in the NHP model [50]. In addition, we and others showed that TBI/PBI with a certain portion of BM shielding (5 to 50% bone marrow) in NHP was associated with maximum structural damage to the intestine characterized by extensive reduction in villous height, loss of crypts, and a massive reduction in epithelial surface area by day 10 [51,52,53]. This is aligned with what we observed in this study, where TBI reduced functional area and villus height in both vehicle and GT3-treated groups largely by day 7, thus suggesting that structural integrity of the intestine was compromised. Importantly, GT3 significantly enhanced crypt depth at day 7 post-irradiation, suggesting that more stem cells survived and proliferated after TBI. Indeed, Liu et al. have shown a strong positive correlation between crypt depth and stem cell survival after irradiation [30]. To our knowledge, this study is the first to report the influence of GT3 on irradiation-induced intestinal structural injuries in NHPs exposed to a supra-lethal dose of total-body radiation. 

The small intestinal epithelium is the fastest dividing tissue in humans with an estimated turnover time of about a week [31]. The intestinal epithelium homeostasis and renewal is attributed to the resident intestinal stem cells located at the bottom of the crypt [34,54]. However, at high doses of radiation, more widespread intestinal apoptosis fails to trigger a regenerative response, which leads to GI failure [32]. Reduction to IR-induced loss of crypt stem cells and the rate of replenishment are crucial factors in determining the extent of jejunal recovery following irradiation [55]. Notably, the jejunum has been shown to be the most radiosensitive region of the small intestine [53]. In the current study even though GT3-treatment did not affect irradiation-induced crypt survival at day 4 or 7, yet we still observed that GT3 showed a trend of increasing crypt survival on day 4. Strikingly, GT3 treatment significantly reduced intestinal cell death in jejunum at both day 4 and 7 post-irradiation. Moreover, Suman et al., showed in a preliminary study using murine model that GT3 promotes intestinal cell survival via upregulation of anti-apoptotic genes or downregulation of pro-apoptotic genes to protect the intestinal cells in response to irradiation [26]. In addition, we observed that GT3 enhanced Ki-67 and PCNA expression in jejunum of irradiated NHPs at day 7 post-irradiation, suggestive of enhanced proliferation status, which may indicate the recovery of intestinal damage after IR. 

Intestinal epithelium is the largest barrier between the internal and external environments and is crucial for protecting the body against toxic molecules that can trigger mucosal inflammation if they cross the barrier [56]. Exposure to radiation causes inflammation and immune imbalance and increases gut permeability [57,58,59]. Intestinal permeability is regulated by intercellular structures termed tight junctions (TJs). The molecular composition of the TJs is complex, consisting of multiple transmembrane proteins including claudin family members, occludin, and members of the junction adhesion molecule (JAM) protein family [60,61], which are disrupted after radiation. We have recently shown that TBI negatively affects TJ-related proteins, which are essential for maintaining gut permeability in the NHP model [37]. Moreover, the increased permeability of GI epithelial cells after radiation exposure frequently results from the redistribution of tight junctions [37,62]. In this study, no significant difference was observed in the expression levels of claudin-2. However, GT3 treatment significantly increased ZO-1 expression levels at day 7. Importantly, ZO-1 has been shown to be critical for mucosal repair [63], and plays a functional role in organizing components of the TJ while linking them to the cortical actin cytoskeleton [64]. There were only small differences noted between the vehicle- and GT3-treated groups for most of the tight junction-related proteins evaluated in the gut at both time points examined (data not shown). This may be related to the relatively small sample size with *N* = 3 (D4) and *N* = 5 (D7). In addition, individual variation within the group could have resulted in diluting the effects induced by GT3.

In summary, we found that GT3 has the ability to regulate progenitor cell survival and cell death in the small intestine. Thus, GT3 may have a protective function in the intestinal epithelial and crypt cells in the context of irradiation-induced damage. Further studies are clearly warranted with increased *N* as GT3 has been shown to be a promising, safe, and non-toxic medical countermeasure for radiation-induced GI injury in the murine model. It will be important to investigate GT3 effects using the partial-body NHP exposure model with LINAC. Such studies will also provide the opportunity to compare the effects of orthovoltage X-rays compared to gamma-rays in the NHP model and investigate the effects of GT3. Studies with GT3 using LINAC as radiation source are in progress.

## 4. Materials and Methods

### 4.1. Animals

A total of 16 naïve rhesus macaques (*Macaca mulatta*, Chinese sub-strain, 6 males and 10 females) were used for this study. These animals were between 3.6–5.5 years of age, weighing 5.45–10.35 kg. The animals were further randomly divided such that eight animals received GT3 (37.5 mg/kg, sc) and the other eight received vehicle. These animals were procured from the National Institutes of Health Animal Center (NIHAC, Poolesville, MD, USA). All animals were maintained in a facility accredited by the Association for Assessment and Accreditation of Laboratory Animal Care (AAALAC)-International. Animals were quarantined for six weeks prior to initiation of the experiment. All NHPs were individually housed in stainless-steel cages in environmentally controlled rooms maintained at 22 °C +/− 2 °C, 30–70% relative humidity, 10–15 air change cycles per h, and a 12:12 h light-dark cycle. Animals were fed a primate diet (Teklad T.2050 diet; Harlan Laboratories Inc., Madison, WI, USA) twice daily with at least six hours between feedings (animals were fed four biscuits each at 7:00 am and 2:00 pm) and received drinking water ad libitum. All the animals received enrichment food (fresh fruits and vegetables, prima treats, peanuts, marshmallows, etc.) once daily Monday–Friday. They received mirrors, toys, and challenge balls for enrichment. Televisions were used for sensory enrichment for 4–5 h at least 3 times a week. All the animals were able to see, hear, and/or touch the conspecifics through the cages. Single housing also eliminated the chance of conflict injuries that could have been caused by pair-housing. Irradiated animals are more prone to infection due to their suppressed immune system. All animals were serologically negative for Macacian herpesvirus 1 (herpes B virus), simian retrovirus (SRV), simian T-cell leukemia virus (STLV), and simian immunodeficiency virus (SIV). They were vaccinated with positive antibody titers for measles and tested negative for tuberculin. Animals were stratified by gender and body weight increases during the quarantine period and then assigned to different treatment groups. All procedures involving animals were approved by the Institutional Animal Care and Use Committee (BIOQUAL Inc.) and the Department of Defense Animal Care and Use Review Office (ACURO). This study was carried out in strict accordance with the recommendations made in the Guide for the Care and Use of Laboratory Animals [65]. 

### 4.2. Experimental Design and Irradiation

The objective of this study was to assess the efficacy of a potential medical countermeasure, GT3, against intestinal injury in a GI-ARS following TBI. Various analyses were performed to understand the mechanism of action of this medical countermeasure in rescuing the GI tract of irradiated macaques. To achieve this objective, a total of 16 animals were used in this study where GT3 was evaluated against a radiation dose of 12.0 Gy ^60^Co gamma-radiation, a supralethal radiation dose capable of inducing GI specific acute radiation syndrome (GI-ARS). The 16 NHPs exposed to 12 Gy were further divided into two groups (eight NHPs each) with one group receiving 37.5 mg/kg GT3 subcutaneously (sc) and the other receiving its vehicle in the dorsal scapular region 24 h prior to radiation exposure (Table 1). In addition to blood collection, these animals were euthanized according to a schedule for tissue collection on various days post-irradiation, and these samples were analyzed for different biomarkers. 

### 4.3. Total-Body Irradiation

On the day before irradiation, NHPs were fasted for approximately 12 h. On the day of irradiation, animals were removed from the transport cages and placed into a standard housing cage in the radiation staging area. Before proceeding with sedation, the unique tattoo identification numbers were confirmed by study personnel. Animals were then sedated in their cages using the squeeze-back mechanism and were administered a 10–15 mg/kg intramuscular (im) dose of ketamine (100 mg/mL) and up to two NHPs were sedated at a time. Once sedated, the animals were placed in restraint boxes to limit movement and to maintain a proper upright sedated position during the irradiation procedure. The NHP limbs were secured to the box using ropes that were tied onto a cleat. Animals were then transported to the High Level Cobalt Facility via elevator, and the NHP tattoo identification numbers were verified again by the attending dosimetrist. A 0.1–0.3 mL im booster of ketamine (100 mg/mL) was administered to the NHPs to limit movement while being irradiated, if needed. For irradiation, two NHPs were placed on the irradiation platform facing away from each other and were exposed to a midline dose (12 Gy) ^60^Co γ-radiation at a dose rate of 0.6 Gy/min from both sides (bilateral, simultaneous exposure) as described earlier [27]. 

To deliver the precise radiation dose, NHP abdominal widths were measured with digital calipers. Animals were observed throughout the irradiation procedure via in-room cameras. After irradiation, the animals were returned to their cages in the housing area and were monitored for recovery from the procedure. The radiation field in the area of the NHP location was uniform within ±1.5%. The dosimetry for photons was based on the alanine/EPR (electron paramagnetic resonance) dosimetry system [66]. This is one of the most precise dosimetry techniques at present which is used by national standards laboratories for the most critical measurements and calibrations. Thus, it is one of the very few methods that are used in regular worldwide inter-comparisons of the national standards of Gy.

### 4.4. Drug Preparation and Administration

GT3 and its vehicle were procured from Callion Pharma (Jonesborough, TN, USA), and supplied as an injectable solution at a concentration of 50 mg/mL. Just prior to administration, the GT3 and vehicle solution were thoroughly mixed using a lab vortex. GT3 or vehicle were administered sc at a dose of 37.5 mg/kg 24 h prior to irradiation. The exact volume administered depended on the individual weight of the NHP. Injections were performed with a sterile 21–25-gauge needle length of ¾–1”. The site for injection (dorsal scapular region [midline]) was prepared as a surgical site before the injection: hair was clipped using # 40 surgical clipper blade and the site was appropriately disinfected to reduce any adverse skin reactions, rash/eruption, inflammation, or irritation. 

### 4.5. Intestinal Tissue Collection

Out of eight NHPs in each group, three were euthanized on day 4 and five on day 7 post-irradiation (see Table 2). Animals scheduled for euthanasia were fasted for 8–12 h before sedation. The NHPs were sedated using ketamine (10–15 mg/Kg, im) and then placed on inhalation anesthesia, 3–5% isoflurane in 100% oxygen (500–1000 cc/min) by mask and maintained with 1–3% isoflurane administered in 100% oxygen (500–1000 cc/min) via endotracheal tube (size 2–4 mm). While the animal was under the anesthesia, vital signs were monitored including SpO_2_, pulse, and body temperature. Upon incision of the abdominal cavity, a 12–14 cm piece of jejunum was isolated and clamped at both ends and surgically removed. After the jejunum was excised from the abdominal cavity, the animal was euthanized, and a full necropsy was performed and additional tissues for further histopathological analysis were collected. 

The segments of proximal jejunum that were procured and fixed in either 10% zinc-buffered formalin or methanol-Carnoy’s (MC) fixative (composition: 60% methanol, 30% chloroform, and 10% glacial acetic acid). These tissue samples were submitted to Histoserv, Inc. (Germantown, MD) for tissue processing and embedding. Slides and paraffin blocks were trimmed from the fixed sections, and these tissues were routinely processed, stained with hematoxylin and eosin (H&E), and submitted for histological and morphometric studies. 

### 4.6. Assessment of TBI-Induced Histological and Morphometric Injury

While the loss of epithelial cells after irradiation shortens the villi, disrupting the structural integrity of the intestine, an increase in crypt depth has been associated with the survival and a higher proliferation rate of stem cells after injury. We assessed the intestinal tissue sections stained with H&E to measure mucosal surface length, villous height, and crypt depth by using a computer assisted image analysis platform. 

#### 4.6.1. Mucosal Surface Length 

A decrease in the surface area of the intestinal mucosa is a sensitive parameter of small bowel radiation injury. Previously, we measured mucosal surface area in mice and rats using a stereologic projection/cycloid method [67,68]. Because of structural differences in the intestine of NHPs (e.g., NHPs have plicae circulares whereas mice and rats do not), we chose computer-assisted Image-Pro^®^ Premier software (Meyer Instruments, Houston, TX, USA) to measure the length of intestinal mucosal surface area in this animal model. All measurements were done at 4X magnification in six to eight areas of intestinal segments. The average of the measurements from those 6–8 areas was used for statistical analyses.

#### 4.6.2. Mucosal Villous Height and Crypt Depth

Mucosal villous height and crypt depth were measured with computer-assisted Image-Pro Premier software. Villous height measurements were conducted on images captured at 4× magnification in six to eight areas of intestinal segments, while crypt depth measurements were conducted on images captured at 10× in eight to ten areas. Mucosal villous height was measured from the tip to the base of each villus, while crypt depth was measured from the crypt base to the tip opening. The average of the measurements from those areas was used for statistical analyses.

### 4.7. Intestinal Crypt Colony Assay

Microcolony crypt cell survival assays were performed as described previously [69,70]. The numbers of surviving and regenerating crypts were analyzed on five H&E stained, transverse sections obtained from proximal jejunum at day 4 and day 7 post-TBI. The H&E slides were scanned at 20× in an Aperio CS2 scanner (Leica Biosystems) and were analyzed through the Imagescope software (Aperio technologies). Surviving crypts (those with ≥10 adjacent, chromophilic, non-Paneth cells) were counted in five circumferences of proximal jejunum per animal, and microcolony survival was expressed as the average number of surviving crypts per circumference. The average from each animal was considered a single value for statistical purposes.

### 4.8. TUNEL Assay

TUNEL assays were performed as described previously [71]. Intestinal tissue sections, which were fixed, dehydrated, and embedded in paraffin and cut at 4 µm thick tissue sections were dewaxed and rehydrated in phosphate-buffered saline. TUNEL assay was performed using an In Situ Cell Death Detection Kit (Roche Diagnostics, Indianapolis, IN, USA) following the manufacturer’s instructions. Intestinal tissue sections were incubated with a reaction mixture of terminal deoxynucleotidyl transferase (TdT) and fluorescein (FITC)-labeled precursor in cacodylate-based buffer for 1 h at 37 °C, rinsed three times with 0.05% Tween-20 in PBS, and counterstained with 4′,6-diamidino-2-phenylindole (DAPI, Invitrogen, Carlsbad, CA, USA) to visualize cell nuclei, mounted under cover slips with Prolong Gold Antifade Mountant (Invitrogen, Carlsbad, CA, USA). TUNEL specificity was controlled by substituting the mixture of TdT and probe with the cacodylate buffer. The green spectrum (FITC) and blue spectrum (DAPI) were used to detect TUNEL-positive cells and nuclei respectively. Images were acquired using the Olympus IX-51 inverted microscope (Olympus America, Center Valley, PA, USA) equipped with Hamamatsu ORCA-ER monochrome camera (Hamamatsu Photonics K.K., Hamamatsu City, Japan). Image analysis was performed using the SlideBook 6.0.15 software. Data were expressed as the percentage of the number of TUNEL-positive nuclei in the total number of nuclei detected by DAPI staining. Any problems with TUNEL image identification were resolved according to the classification of TUNEL images described in the recent review [72].

### 4.9. Fluorescent Microscopy

The immunostaining of NHP intestine sections was performed as described previously [73]. Briefly, 4-µm thick tissue sections were dewaxed and rehydrated in phosphate buffered saline. These intestine sections were then immunostained with rabbit anti-claudin-2 (1:100), rabbit anti-occludin (1:500), rabbit anti-β-catenin (1:2000) (all from cell signaling technology, Danvers, MA); rat anti-Ki-67 (1:200), mouse anti-Zona-1(1:450); mouse anti-claudin-7 (1:800), and mouse anti-E-cadherin (1:2500) (all from Invitrogen, Carlsbad, CA, USA) in blocking buffer (1% bovine serum albumin, 10% normal donkey serum/goat serum, 0.012% saponin, 0.05% Tween-20). The primary antibodies were either detected with donkey anti-rabbit/rat secondary IgG-AlexaFluor 594 conjugates/donkey anti-mouse AlexaFluor 488 conjugate/goat anti-rabbit AlexaFluor 488 conjugate/goat anti-mouse AlexaFluor 594 conjugate (1:800, Invitrogen, Carlsbad, CA, USA). Control staining was performed by substituting the blocking buffer containing primary antibody with blocking buffer only. Tissue sections were counterstained with 4′,6-diamidino-2-phenylindole (DAPI, Thermo Fisher, Waltham, MA, USA) to visualize cell nuclei, mounted under cover slips with Prolong^®^ Gold Antifade Mountant (Invitrogen, Carlsbad, CA, USA). Images were captured using the Olympus IX-51 inverted microscope (Olympus America, Center Valley, PA, USA) equipped with Hamamatsu ORCA-ER monochrome camera (Hamamatsu Photonics K.K., Hamamatsu City, Japan). Image analysis was performed using the Slide Book 6.0.15 software. For quantification, 10 independent fields of view were collected per each NHP intestine section, and for Ki-67 staining, data were presented as the percentage of the number of Ki-67-positive nuclei, while for others, mean optical density (MOD) was recorded for each of the channels used above. The data were presented as an average of MOD/field of view for each channel.

### 4.10. Immunohistochemistry 

Immunohistochemical staining was performed with standard techniques using an avidin–biotin complex, diaminobenzidine chromogen, and hematoxylin counterstaining. Monoclonal mouse anti-proliferating cell nuclear antigen antibody (clone PC-10, EMD Millipore, Billerica, MA, USA) was employed to detect PCNA expression on the proximal jejunum sections obtained at day 4 and 7 post-irradiation (with and without GT3). Human and rhesus macaques (*Macaca mulatta*) PCNA share 100% amino acid sequence homology in the target epitope region (amino acids 112–121). This level of homology allows for sufficient cross-reactivity of clone PC10 with PCNA of the NHP and for a valid immunohistochemistry assay. Sections were deparaffinized, rehydrated and endogenous peroxidase was blocked with 1% H2O2 in methanol for 30 min at room temperature. Nonspecific binding was reduced with 10% normal goat serum (Vector Laboratories, Burlingame, CA, USA) in 3% dry powdered milk in tris–buffered saline (TBS) for 30 min. Sections were incubated with mouse anti-PCNA (1:200, 2 h, EMD Millipore). This was followed by a 30-min incubation with the biotinylated goat anti-mouse IgG (1:400, Vector Laboratories). Sections were further incubated with avidin–biotin–peroxidase complex for 45 min (1:100, Vector Laboratories). Peroxidase binding was visualized with 0.5 mg/mL 3,3-diaminobenzidine tetrahydrochloride solution (Sigma-Aldrich, St. Louis, MO, USA) and 0.003% H2O2 in TBS. Slides were then rinsed with distilled water, counterstained with Harris’s hematoxylin, dehydrated, and cover slipped. PCNA stained slides were scanned using an Aperio Scanner CS2 at 20×. 

### 4.11. Statistical Analysis

Statistical analyses were performed using GraphPad Prism Version 9.1.0 (GraphPad Software, San Diego, CA, USA). Multiple means were compared by *ANOVA* and pairwise comparisons were analyzed with the student’s *t*-test. A value of *p* < 0.05 was considered statistically significant. 

## Figures and Tables

**Table 1:** Experimental study design for 16 NHPs exposed to 12 Gy TBI.

Study Design with 16 NHPs					
GI Study (Total-Body Irradiation, ^60^Co γ-Radiation, 0.6 Gy/min)					
NHP	Drug	Route	Dose	Frequency	Irradiation Dose (Gy)
8 (3M/5F)	GT3	sc	37.5 mg/kg	24 h prior to irradiation	12
8 (3M/5F)	Veh	sc	37.5 mg/kg	24 h prior to irradiation	12

**Table 2:** Euthanasia schedule for 16 NHPs exposed to 12 Gy TBI for GI injury.

Euthanasia Schedule for GI Injury		
Groups	Day 4 Post-Irradiation	Day 7 Post-Irradiation
GT3 + 12 Gy	3	5
Vehicle + 12 Gy	3	5

## Data Availability

All relevant data, which supports the findings of the study, are within the manuscript.

## References

[1] Gosden C., Gardener D. (2005). Weapons of mass destruction—Threats and responses. BMJ.

[2] Singh V.K., Santiago P.T., MacVittie T.J. (2018). Opportunities and challenges with animal models for acute radiation syndrome drug discovery. Expert Opin. Drug Discov..

[3] Williams J.P., Brown S.L., Georges G.E., Hauer-Jensen M., Hill R.P., Huser A.K., Kirsch D.G., Macvittie T.J., Mason K.A., Medhora M.M. (2010). Animal models for medical countermeasures to radiation exposure. Radiat. Res..

[4] Singh V.K., Newman V.L., Berg A.N., MacVittie T.J. (2015). Animal models for acute radiation syndrome drug discovery. Expert Opin. Drug Discov..

[5] Potten C.S. (1990). A Comprehensive Study of the Radiobiological Response of the Murine (BDF1) Small Intestine. Int. J. Radiat. Biol..

[6] Vigneulle R.M., Rao S., Fasano A., MacVittie T.J. (2002). Structural and functional alterations of the gastrointestinal tract following radiation-induced injury in the rhesus monkey. Dig. Dis. Sci..

[7] Chiba M., Uehara H., Niiyama I., Kuwata H., Monzen S. (2020). Changes in miRNA expressions in the injured small intestine of mice following highdose radiation exposure. Mol. Med. Rep..

[8] Denham J.W., Hauer-Jensen M., Peters L.J. (2001). Is it time for a new formalism to categorize normal tissue radiation injury?. Int. J. Radiat. Oncol. Biol. Phys..

[9] Booth C., Potten C.S. (2002). The Intestine as a Model for Studying Stem-Cell Behavior.

[10] Withers H.R., Elkind M.M. (1968). Dose-survival characteristics of epithelial cells of mouse intestinal mucosa. Radiology.

[11] Rios C.I., Cassatt D.R., Dicarlo A.L., Macchiarini F., Ramakrishnan N., Norman M.K., Maidment B.W. (2014). Building the strategic national stockpile through the NIAID Radiation Nuclear Countermeasures Program. Drug Dev. Res..

[12] Singh V.K., Hauer-Jensen M. (2016). Gamma-Tocotrienol as a Promising Countermeasure for Acute Radiation Syndrome: Current Status. Int. J. Mol. Sci..

[13] Singh V.K., Seed T.M. (2021). Radiation countermeasures for hematopoietic acute radiation syndrome: Growth factors, cytokines and beyond. Int. J. Radiat. Biol..

[14] Sailo B.L., Banik K., Padmavathi G., Javadi M., Bordoloi D., Kunnumakkara A.B. (2018). Tocotrienols: The promising analogues of vitamin E for cancer therapeutics. Pharmacol. Res..

[15] Singh V.K., Beattie L.A., Seed T.M. (2013). Vitamin E: Tocopherols and tocotrienols as potential radiation countermeasures. J. Radiat. Res..

[16] Nesaretnam K. (2008). Multitargeted therapy of cancer by tocotrienols. Cancer Lett..

[17] Parker R.A., Pearce B.C., Clark R.W., Gordon D.A., Wright J.J. (1993). Tocotrienols regulate cholesterol production in mammalian cells by post-transcriptional suppression of 3-hydroxy-3-methylglutaryl-coenzyme A reductase. J. Biol. Chem..

[18] Song B.L., DeBose-Boyd R.A. (2006). Insig-dependent ubiquitination and degradation of 3-hydroxy-3-methylglutaryl coenzyme a reductase stimulated by delta- and gamma-tocotrienols. J. Biol. Chem..

[19] Theriault A., Chao J.T., Gapor A. (2002). Tocotrienol is the most effective vitamin E for reducing endothelial expression of adhesion molecules and adhesion to monocytes. Atherosclerosis.

[20] Ghosh S.P., Kulkarni S., Hieber K., Toles R., Romanyukha L., Kao T.C., Hauer-Jensen M., Kumar K.S. (2009). Gamma-tocotrienol, a tocol antioxidant as a potent radioprotector. Int. J. Radiat. Biol..

[21] Berbee M., Fu Q., Boerma M., Wang J., Kumar K.S., Hauer-Jensen M. (2009). gamma-Tocotrienol ameliorates intestinal radiation injury and reduces vascular oxidative stress after total-body irradiation by an HMG-CoA reductase-dependent mechanism. Radiat. Res..

[22] Garg S., Sadhukhan R., Banerjee S., Savenka A.V., Basnakian A.G., McHargue V., Wang J., Pawar S.A., Ghosh S.P., Ware J. (2019). Gamma-Tocotrienol Protects the Intestine from Radiation Potentially by Accelerating Mesenchymal Immune Cell Recovery. Antioxidants.

[23] Kulkarni S.S., Cary L.H., Gambles K., Hauer-Jensen M., Kumar K.S., Ghosh S.P. (2012). Gamma-tocotrienol, a radiation prophylaxis agent, induces high levels of granulocyte colony-stimulating factor. Int. Immunopharmacol..

[24] Kulkarni S., Singh P.K., Ghosh S.P., Posarac A., Singh V.K. (2013). Granulocyte colony-stimulating factor antibody abrogates radioprotective efficacy of gamma-tocotrienol, a promising radiation countermeasure. Cytokine.

[25] Singh V.K., Wise S.Y., Fatanmi O.O., Scott J., Romaine P.L., Newman V.L., Verma A., Elliott T.B., Seed T.M. (2014). Progenitors mobilized by gamma-tocotrienol as an effective radiation countermeasure. PLoS ONE.

[26] Suman S., Datta K., Chakraborty K., Kulkarni S.S., Doiron K., Fornace A.J., Sree Kumar K., Hauer-Jensen M., Ghosh S.P. (2013). Gamma tocotrienol, a potent radioprotector, preferentially upregulates expression of anti-apoptotic genes to promote intestinal cell survival. Food Chem. Toxicol..

[27] Singh V.K., Kulkarni S., Fatanmi O.O., Wise S.Y., Newman V.L., Romaine P.L., Hendrickson H., Gulani J., Ghosh S.P., Kumar K.S. (2016). Radioprotective Efficacy of Gamma-Tocotrienol in Nonhuman Primates. Radiat. Res..

[28] Fendler W., Malachowska B., Meghani K., Konstantinopoulos P.A., Guha C., Singh V.K., Chowdhury D. (2017). Evolutionarily conserved serum microRNAs predict radiation-induced fatality in nonhuman primates. Sci. Transl. Med..

[29] Rosen E., Fatanmi O.O., Wise S.Y., Rao V.A., Singh V.K. (2022). Gamma-tocotrienol, a radiation countermeasure, reverses proteomic changes in serum following total-body gamma irradiation in mice. Sci. Rep..

[30] Liu Z., Tian H., Jiang J., Yang Y., Tan S., Lin X., Liu H., Wu B. (2016). Beta-Arrestin-2 modulates radiation-induced intestinal crypt progenitor/stem cell injury. Cell Death Differ..

[31] Beumer J., Clevers H. (2021). Cell fate specification and differentiation in the adult mammalian intestine. Nat. Rev. Mol. Cell Biol..

[32] Potten C.S. (2004). Radiation, the ideal cytotoxic agent for studying the cell biology of tissues such as the small intestine. Radiat. Res..

[33] Booth D., Potten C.S. (2001). Protection against mucosal injury by growth factors and cytokines. J. Natl. Cancer Inst. Monogr..

[34] Potten C.S., Booth C., Pritchard D.M. (1997). The intestinal epithelial stem cell: The mucosal governor. Int. J. Exp. Pathol..

[35] Celis J.E., Bravo R., Larsen P.M., Fey S.J. (1984). Cyclin: A nuclear protein whose level correlates directly with the proliferative state of normal as well as transformed cells. Leuk. Res..

[36] Gerdes J., Li L., Schlueter C., Duchrow M., Wohlenberg C., Gerlach C., Stahmer I., Kloth S., Brandt E., Flad H.D. (1991). Immunobiochemical and molecular biologic characterization of the cell proliferation-associated nuclear antigen that is defined by monoclonal antibody Ki-67. Am. J. Pathol..

[37] Garg S., Zheng J., Wang J., Authier S., Pouliot M., Hauer-Jensen M. (2016). Segmental Differences in Radiation-Induced Alterations of Tight Junction-Related Proteins in Non-Human Primate Jejunum, Ileum and Colon. Radiat. Res..

[38] Dorr H., Meineke V. (2011). Acute radiation syndrome caused by accidental radiation exposure—Therapeutic principles. BMC Med..

[39] VandeBerg J.L., Williams-Blangero S. (1997). Advantages and limitations of nonhuman primates as animal models in genetic research on complex diseases. J. Med. Primatol..

[40] Uno Y., Uehara S., Yamazaki H. (2016). Utility of non-human primates in drug development: Comparison of non-human primate and human drug-metabolizing cytochrome P450 enzymes. Biochem. Pharmacol..

[41] Singh V.K., Olabisi A.O. (2017). Nonhuman primates as models for the discovery and development of radiation countermeasures. Expert Opin. Drug Discov..

[42] Cheema A.K., Mehta K.Y., Fatanmi O.O., Wise S.Y., Hinzman C.P., Wolff J., Singh V.K. (2017). A Metabolomic and Lipidomic Serum Signature from Nonhuman Primates Administered with a Promising Radiation Countermeasure, Gamma-Tocotrienol. Int. J. Mol. Sci..

[43] Palozza P., Verdecchia S., Avanzi L., Vertuani S., Serini S., Iannone A., Manfredini S. (2006). Comparative antioxidant activity of tocotrienols and the novel chromanyl-polyisoprenyl molecule FeAox-6 in isolated membranes and intact cells. Mol. Cell. Biochem..

[44] Shea-Donohue T., Fasano A., Zhao A., Notari L., Yan S., Sun R., Bohl J.A., Desai N., Tudor G., Morimoto M. (2016). Mechanisms Involved in the Development of the Chronic Gastrointestinal Syndrome in Nonhuman Primates after Total-Body Irradiation with Bone Marrow Shielding. Radiat. Res..

[45] Fu Q., Berbee M., Wang W., Boerma M., Wang J., Schmid H.A., Hauer-Jensen M. (2011). Preclinical evaluation of Som230 as a radiation mitigator in a mouse model: Postexposure time window and mechanisms of action. Radiat. Res..

[46] Hauer-Jensen M., Poulakos L., Osborne J.W. (1988). Effects of accelerated fractionation on radiation injury of the small intestine: A new rat model. Int. J. Radiat. Oncol. Biol. Phys..

[47] Booth C., Tudor G., Tudor J., Katz B.P., MacVittie T.J. (2012). Acute gastrointestinal syndrome in high-dose irradiated mice. Health Phys..

[48] MacVittie T.J., Bennett A.W., Farese A.M., Taylor-Howell C., Smith C.P., Gibbs A.M., Prado K., Jackson W. (2015). The Effect of Radiation Dose and Variation in Neupogen(R) Initiation Schedule on the Mitigation of Myelosuppression during the Concomitant GI-ARS and H-ARS in a Nonhuman Primate Model of High-dose Exposure with Marrow Sparing. Health Phys..

[49] MacVittie T.J., Farese A.M., Parker G.A., Jackson W., Booth C., Tudor G.L., Hankey K.G., Potten C.S. (2019). The Gastrointestinal Subsyndrome of the Acute Radiation Syndrome in Rhesus Macaques: A Systematic Review of the Lethal Dose-response Relationship with and without Medical Management. Health Phys..

[50] Wang J., Shao L., Hendrickson H.P., Liu L., Chang J., Luo Y., Seng J., Pouliot M., Authier S., Zhou D. (2015). Total Body Irradiation in the "Hematopoietic" Dose Range Induces Substantial Intestinal Injury in Non-Human Primates. Radiat. Res..

[51] MacVittie T.J., Bennett A., Booth C., Garofalo M., Tudor G., Ward A., Shea-Donohue T., Gelfond D., McFarland E., Jackson W. (2012). The prolonged gastrointestinal syndrome in rhesus macaques: The relationship between gastrointestinal, hematopoietic, and delayed multi-organ sequelae following acute, potentially lethal, partial-body irradiation. Health Phys..

[52] Jones J.W., Bennett A., Carter C.L., Tudor G., Hankey K.G., Farese A.M., Booth C., MacVittie T.J., Kane M.A. (2015). Citrulline as a Biomarker in the Non-human Primate Total- and Partial-body Irradiation Models: Correlation of Circulating Citrulline to Acute and Prolonged Gastrointestinal Injury. Health Phys..

[53] Wang J., Garg S., Landes R.D., Liu L., Fu Q., Seng J., Boerma M., Thrall K., Hauer-Jensen M., Pathak R. (2021). Differential Recovery of Small Intestinal Segments after Partial-Body Irradiation in Non-Human Primates. Radiat. Res..

[54] Booth C., Potten C.S. (2000). Gut instincts: Thoughts on intestinal epithelial stem cells. J. Clin. Invest..

[55] Takemura N., Kawasaki T., Kunisawa J., Sato S., Lamichhane A., Kobiyama K., Aoshi T., Ito J., Mizuguchi K., Karuppuchamy T. (2014). Blockade of TLR3 protects mice from lethal radiation-induced gastrointestinal syndrome. Nat. Commun..

[56] Cario E. (2008). Barrier-protective function of intestinal epithelial Toll-like receptor 2. Mucosal. Immunol..

[57] Gremy O., Benderitter M., Linard C. (2008). Acute and persisting Th2-like immune response after fractionated colorectal gamma-irradiation. World J. Gastroenterol..

[58] Turner J.R. (2009). Intestinal mucosal barrier function in health and disease. Nat. Rev. Immunol..

[59] Garg S., Boerma M., Wang J., Fu Q., Loose D.S., Kumar K.S., Hauer-Jensen M. (2010). Influence of sublethal total-body irradiation on immune cell populations in the intestinal mucosa. Radiat. Res..

[60] Suzuki T. (2013). Regulation of intestinal epithelial permeability by tight junctions. Cell Mol. Life Sci..

[61] Rowlands B.J., Soong C.V., Gardiner K.R. (1999). The gastrointestinal tract as a barrier in sepsis. Br. Med. Bull..

[62] Shukla P.K., Gangwar R., Manda B., Meena A.S., Yadav N., Szabo E., Balogh A., Lee S.C., Tigyi G., Rao R. (2016). Rapid disruption of intestinal epithelial tight junction and barrier dysfunction by ionizing radiation in mouse colon in vivo: Protection by N-acetyl-l-cysteine. Am. J. Physiol. Gastrointest. Liver Physiol..

[63] Kuo W.T., Zuo L., Odenwald M.A., Madha S., Singh G., Gurniak C.B., Abraham C., Turner J.R. (2021). The Tight Junction Protein ZO-1 Is Dispensable for Barrier Function but Critical for Effective Mucosal Repair. Gastroenterology.

[64] Fanning A.S., Jameson B.J., Jesaitis L.A., Anderson J.M. (1998). The tight junction protein ZO-1 establishes a link between the transmembrane protein occludin and the actin cytoskeleton. J. Biol. Chem..

[65] National Research Council of the National Academy of Sciences (2011). Guide for the Care and Use of Laboratory Animals.

[66] Nagy V. (2000). Accuracy considerations in EPR dosimetry. Appl. Radiat. Isot..

[67] Baddeley A.J., Gundersen H.J., Cruz-Orive L.M. (1986). Estimation of surface area from vertical sections. J. Microsc..

[68] Langberg C.W., Sauer T., Reitan J.B., Hauer-Jensen M. (1996). Relationship between intestinal fibrosis and histopathologic and morphometric changes in consequential and late radiation enteropathy. Acta Oncol..

[69] Hendry J.H., Potten C.S., Roberts N.P. (1983). The gastrointestinal syndrome and mucosal clonogenic cells: Relationships between target cell sensitivities, LD50 and cell survival, and their modification by antibiotics. Radiat. Res..

[70] Withers H.R., Elkind M.M. (1970). Microcolony survival assay for cells of mouse intestinal mucosa exposed to radiation. Int. J. Radiat. Biol. Relat. Stud. Phys. Chem. Med..

[71] Apostolov E.O., Wang X., Shah S.V., Basnakian A.G. (2007). Role of EndoG in development and cell injury. Cell Death Differ..

[72] Moore C.L., Savenka A.V., Basnakian A.G. (2021). TUNEL Assay: A Powerful Tool for Kidney Injury Evaluation. Int. J. Mol. Sci..

[73] Singh M., Odeniyi D.T., Apostolov E.O., Savenka A., Fite T., Wangila G.W., Walker R.B., Basnakian A.G. (2013). Protective effect of zinc-N-acetylcysteine on the rat kidney during cold storage. Am. J. Physiol. Renal. Physiol..

